# A comparative study between central quadrantectomy and nipple resection with areola preservation Versus Grisotti flap mammoplasty in central breast lesions extending to nipple: a randomized clinical trial

**DOI:** 10.1186/s43046-024-00253-z

**Published:** 2025-01-20

**Authors:** Philobater Bahgat Adly Awad, Basma Hussein Abdelaziz Hassan, Abanoub Adel Shafek Awad, Abdelrahman Ahmed Younis Mohamed Attaia, Kerolos Bahgat Adly Awad, Dina Mohamed Hanafy, Ahmed Gamal El Din Osman

**Affiliations:** General Surgery Department, Faculty of Medicine, Ain Shams University, Cairo, Egypt

**Keywords:** Breast cancer, Oncoplastic breast surgery, Grisotti flap, Mammoplasty, Central breast tumors, Grisotti technique

## Abstract

**Objectives:**

To evaluate central quadrantectomy and nipple resection with areola preservation (CQ-NR-AP) as a new reconstructive oncoplastic technique Versus Grisotti flap mammoplasty (GFM) in central malignant tumors of the breast extending to the nipple, in terms of time procedures, breast symmetry, patient satisfaction, postoperative complications, and local recurrence.

**Patients and methods:**

The current study is a single-blind, single-center, randomized, controlled trial that was performed between May 2018 and May 2023 in the breast surgery unit of University Hospitals. This trial involved 40 individuals who had central breast lesions that extended to the nipple and were monitored for two years following surgery.

**Results:**

As regards the mean intra-operative time in minutes, in the group (I) was 80.1 with a standard deviation of ± 13.9, and ingroup (II) was 138.9 with a standard deviation of ± 14.02 (*p* = 0.001). The seroma was detected in zero cases in group (I) and 2(10%) cases in group II (*p* = 0.487) and those two cases were managed by aspiration only. Regarding, the wound infection was found in one case (5%) in group (I) and 3(15%) cases in group II (*p* = 0.605). Regarding patient satisfaction and breast, symmetry was much better in the group (I).

**Conclusion:**

The safety and ease of central quadrantectomy and nipple resection with areola preservation were demonstrated in a two-year follow-up, with a lower incidence of complications compared to the Grisotti flap mammoplasty technique. Furthermore, this approach was associated with higher patient satisfaction, which is a significant achievement in the management of centrally located breast tumors.

**Trial registration:**

PACTR202405688323721. 28/05/2024.

## Introduction

Breast cancer is the most common cancer in women [1] and is currently one of the most frequently diagnosed cancers and the fifth cause of cancer-related deaths with an estimated number of 2.3 million new cases worldwide according to the GLOBOCAN 2020 data [2].

According to the literature, tumors located in the retro-areolar region account for 5 to 20% of breast cancer [3, 4].

Patients with centrally located breast cancer were used to receive radical mastectomy because of safety and cosmetic problems, but recently breast-conserving therapy in combination with postoperative radiotherapy has been shown to have an equivalent survival rate to mastectomy and has emerged as the most effective method of treating breast cancer [5].

The development of oncoplastic surgery in recent years has allowed for an improvement in the treatment of breast cancers. It maintains the same level of oncological safety as radical surgery while enabling the performance of a massive glandular excision with a satisfactory aesthetic outcome.

In 1993, Galimberti et al. described an oncoplastic procedure (Grisotti flap) for the management of retro-areolar breast cancers. He described the technique as excision of the NAC, directly over the site of the tumor, extending down to mobilize a dermo-glandular flap which is then de-epithelized in order to reshape the breast and recreate an areola [6].

In tumors involving lactiferous ducts but 1 cm away from the areola, we tried to perform nipplectomy with areola preservation and central quadrantectomy to avoid losing the normal color and sensation of areola and keep the normal anatomical configuration of the breast.

In our study, we will compare the Grisotti technique versus nipplectomy and central quadrantectomy with areola preservation, as in malignancies extending to the nipple, more than 1 cm away from the areola.

### Aim of work

To compare nipplectomy and central quadrantectomy with areola preservation as a new reconstructive oncoplastic technique Versus Grisotti flap mammoplasty in central malignant tumors of the breast extending to the nipple, in terms of time procedures, breast symmetry, patient satisfaction, postoperative complications, and local recurrence.

### Patients and methods

The present study was a single-blind, prospective, randomized, controlled single-center trial that was conducted at University Hospitals' breast surgical unit from May 2018 to May 2023. This trial followed the CONSORT guidelines and involved 40 female patients who had centrally located breast carcinoma extending to the nipple. Twenty patients underwent nipplectomy and central quadrantectomy with areola preservation, and the other 20 patients underwent Grisotti flap mammoplasty. The citations, references, and in-line citations were not modified, and the American English spelling, specific terms, and phrases were strictly adhered to.

#### Inclusion criteria

1. Female Patients above 18
2. Patients with proven breast cancer involving lactiferous ducts but at least 1 cm away from the areola.

#### Exclusion criteria

1. Candidates with ages below 18 years old.
2. Patients with proven breast cancer involving lactiferous ducts less than 1 cm away from the areola Patient refusal.

All individuals (as shown in Table 1) were initially evaluated during the preoperative period, following the confirmation of their diagnosis via biopsy. A multidisciplinary team consisting of a surgeon, oncologist, pathologist, and radiation therapist determined whether to proceed with upfront surgery or neoadjuvant therapy. The same surgeon performed all operations. For each case, the status of the lymph nodes was assessed through either a complete lymph node dissection or a sentinel lymph node biopsy with frozen section analysis. Depending on the lymph node status, a complete axillary lymph node dissection might be performed. After tumor resection in the central quadrant, an intraoperative frozen section analysis is conducted to verify clear margins.

**Table 1:** Detailed descriptions of patient tumor characteristics

Patient	Age (yr)	Type of tumor	Size of Tumor (mm)	Tumor Stage	Grade	SLN Status	Surgical Technique	Adjuvant Therapy
1	54	Lobular	17	1c	2	positive	Grisotti	RT,HT
2	79	Ductal	19	1c	3	positive	CQ-NR-AP	RT, HT
3	81	DCIS	33	INSITU	2	Negative	CQ-NR-AP	None,RT
4	49	Ductal	7	1b	1	Negative	CQ-NR-AP	None
5	56	Ductal	13	1c	3	Negative	Grisotti	RT, CT, HT
6	65	Ductal	14	1c	3	Positive	Grisotti	RT,HT
7	74	Ductal	15	1c	3	Positive	Grisotti	RT,HT
8	46	Mucinous	15	1c	2	Positive	CQ-NR-AP	RT, HT
9	50	Lobular	15	1c	2	Negative	CQ-NR-AP	RT, HT
10	75	Lobular	17	1c	2	Positive	CQ-NR-AP	RT, HT
11	74	Paget	34	INSITU	1	Negative	Grisotti	RT,HT
12	62	DCIS	49	INSITU	2	Negative	CQ-NR-AP	None,RT
13	55	DCIS	30	INSITU	1	Negative	CQ-NR-AP	None,RT
14	42	Ductal	18	1c	2	Positive	Grisotti	RT,HT
15	61	Ductal	14	1c	2	Positive	Grisotti	HT,RT
16	42	Lobular	16	1c	3	Positive	Grisotti	RT,HT
17	46	DCIS	50	INSITU	2	Negative	CQ-NR-AP	None,RT
18	74	Ductal	12	1c	2	Positive	Grisotti	HT,RT
19	69	Ductal	14	1c	2	Positive	Grisotti	RT,HT
20	42	Ductal	17	1c	2	Positive	CQ-NR-AP	RT, HT
21	42	Lobular	19	1c	3	Positive	Grisotti	RT,HT
22	55	Mucinous	13	1c	1	Negative	Grisotti	None
23	49	Ductal	24	2	3	Positive	CQ-NR-AP	RT, CT, HT
24	58	Ductal	9	1b	2	Negative	Grisotti	None, RT
25	47	Ductal	7	1b	1	Negative	CQ-NR-AP	None
26	42	DCIS	31	INSITU	2	Negative	Grisotti	None,RT
27	51	papillary	8	1b	2	Negative	CQ-NR-AP	None
28	71	Ductal	17	1c	3	Positive	Grisotti	RT,CT,HT
29	63	Lobular	22	2	3	positive	CQ-NR-AP	RT, CT, HT
30	44	Ductal	10	1b	2	Negative	CQ-NR-AP	RT, HT
31	52	Ductal	15	2	1	Negative	Grisotti	None, RT
32	66	Ductal	17	1b	2	Negative	CQ-NR-AP	None, RT
33	42	Ductal	34	1b	1	Negative	CQ-NR-AP	RT, HT
34	42	Lobular	49	INSITU	2	Positive	Grisotti	HT,RT
35	55	Mucinous	30	INSITU	2	Positive	Grisotti	RT,HT
36	66	DCIS	18	1c	3	Positive	Grisotti	None,RT
37	58	Ductal	14	1c	2	Negative	CQ-NR-AP	HT, RT
38	47	Ductal	15	1c	2	Positive	Grisotti	RT,HT
39	43	Ductal	17	INSITU		Positive	CQ-NR-AP	RT, CT, HT
40	52	Lobular	34	1c		Negative	Grisotti	None,RT

*SLN* sentinel lymph node, *IBRM* inferiorly based reduction mammoplasty, *RT* radiotherapy, *HT* hormone therapy, *DCIS* ductal carcinoma in situ, *CT* chemotherapy

t These two patients developed distant metastasis. None of the patients showed angiolymphatic tumor invasion

### Randomization and blinding

The randomization process was carried out the day before surgery, and patients were assigned to either experimental Group I for nipplectomy and central quadrantectomy with areola preservation or experimental Group II for Grisotti flap mammoplasty using a computer-generated randomization code. The two groups were balanced at a ratio of 1:1 to ensure that the study was conducted under single-blind conditions. This study was a randomized control trial of parallel design, with a sample size of 20 patients in each group, resulting in a total sample size of 40 patients. The sample size was estimated using the online software "Riskcalc®" and the study was a non-inferiority clinical trial with a least allowable clinical difference of 15%. The study patients were included and randomized via block randomization, where the first twenty-eight patients who met the eligibility criteria underwent axillary lymph node ultrasound-shear dissection, while the next twenty-eight patients underwent conventional radio frequency electric dissection.

### Pre-operative

#### The pre-operative investigations

Laboratory tests, including a routine complete blood count, liver and kidney profiles, coagulation profile, blood sugar level, and comprehensive virology screening, were performed. Additionally, bilateral mammography and ultrasound exams (for breast and axillary exploration) were conducted, with optional bilateral breast magnetic resonance imaging (MRI) if necessary. Biopsies were taken from the tumor, followed by histological and immunohistochemical analysis, including the examination of hormone receptors, Ki67 proliferation index, and HER2 status. ECG and echocardiography were performed upon request by the anesthesiologist when indicated, along with stress ECG. Finally, patient counseling and consent were obtained.

#### Operative details

All surgical procedures were carried out by a single team under general anesthesia. Prior to the start of the operation, all patients received a single dose of 1g of a third-generation cephalosporin intravenously. The breast surgery team, consisting of multiple specialties, discussed and planned the surgical scar resection area, tumor size and location, and axillary dissection. The reduction pattern and tumor location were marked on the patient's standing breast, followed by prepping and draping. The operation began with a sentinel lymph node biopsy in the ipsilateral axilla, which was subjected to frozen section analysis. The tumor was then removed, weighed, and sent for mammographic and pathological evaluation. After the tumor was removed, tissue extensions were taken from all tumor bed dimensions, and surgical clips were used to mark the tumor bed margins to aid in the location of the original tumor bed for the expected radiation boost. The contralateral breast underwent adjustment using the superior-medial pattern of breast reduction. Finally, the patient was positioned upright for the final assessment of symmetry, flap placement, and breast shape. Drainage was assessed in each breast using the oncoplastic technique, and if necessary, two drains were inserted (one in the contralateral breast and one in the axilla after axillary dissection). The incisions were then sutured, with suturing done in stages to ensure that the deeper glandular layers were positioned as far forward as possible to completely fill the central defect and create a satisfactory cone. After the subcutaneous layers were closed, cutaneous closure was achieved using continuous intradermal sutures. The surgical scars were then covered with strips and gauze pads, and a comfortable sports bra was fitted over the entire operated field.

##### Group (I) underwent nipplectomy and central quadrantectomy with areola preservation

**Fig. 1:**
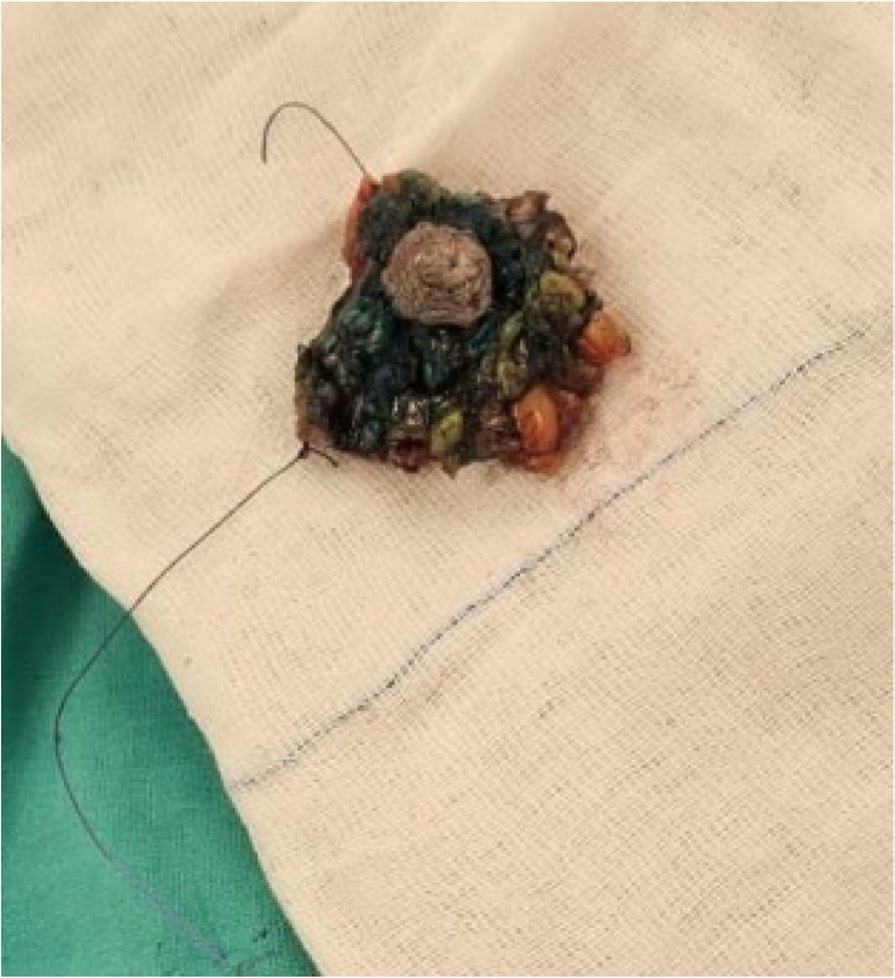
Breast excised biopsy

**Fig. 2:**
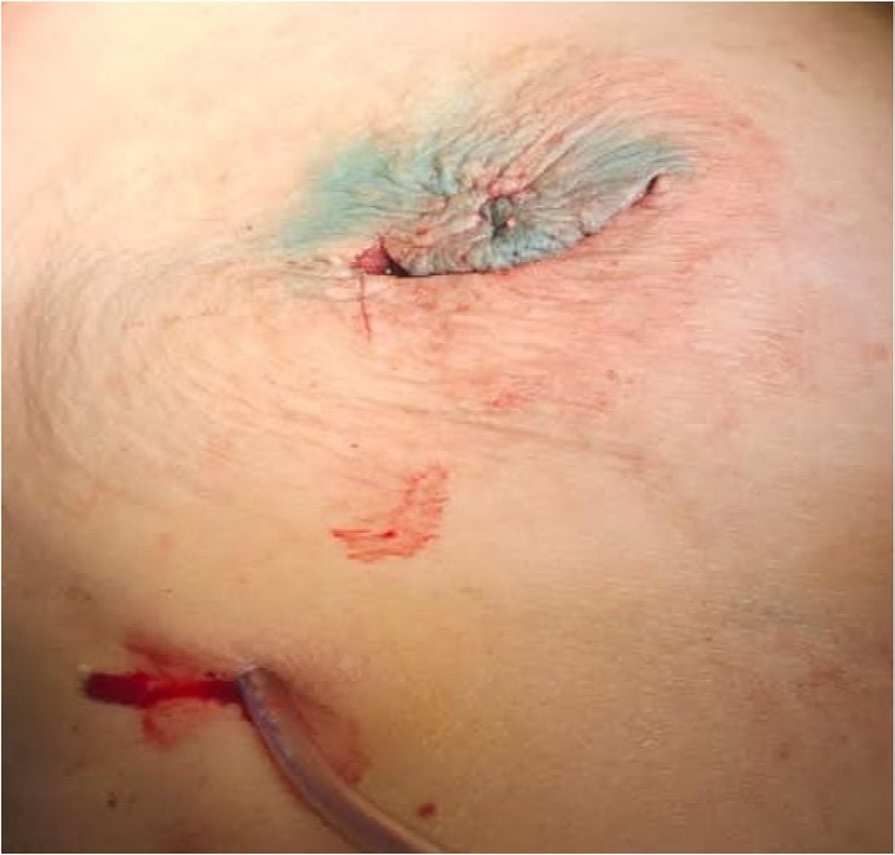
After closing the subcutaneous layers in nipplectomy and central quadrantectomy with areola preservation with the drain

- A periareolar round incision and a cut around the nipple were used to perform a wire-guided lumpectomy, reaching the depth of the resected breast cone tissue and preserving the areola. Figure (1).
- After the tumor had been removed, the orientation of the breast defect determined the positioning of the glandular pedicle and the tissue rearrangement. The pedicle stub was obtained along with additional glandular tissue from the remaining breast, which was then repositioned in the central area of the breast to achieve better projection. Fig (2).

##### Group (II) underwent Grisotti flap mammoplasty

**Fig. 3:**
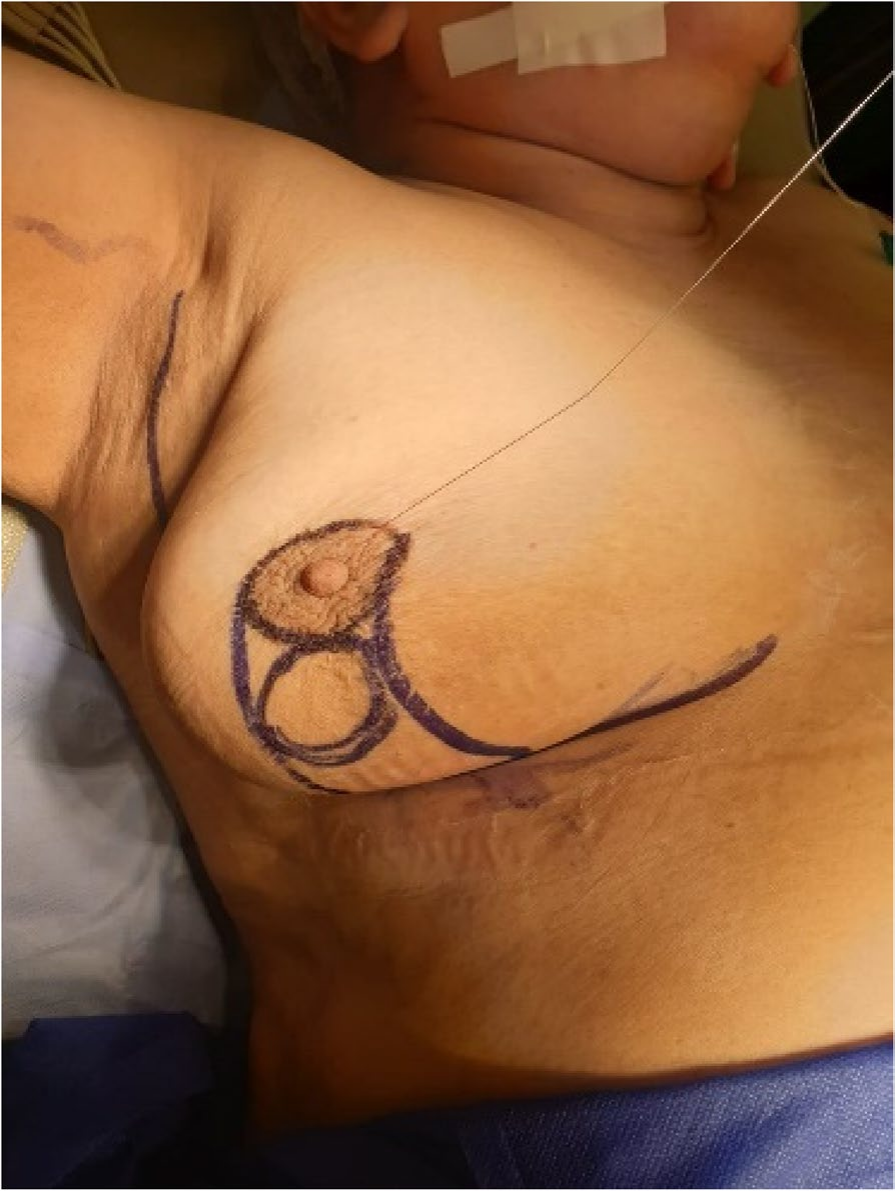
Marking breast for Grisotti Flap mammoplasty

**Fig. 4:**
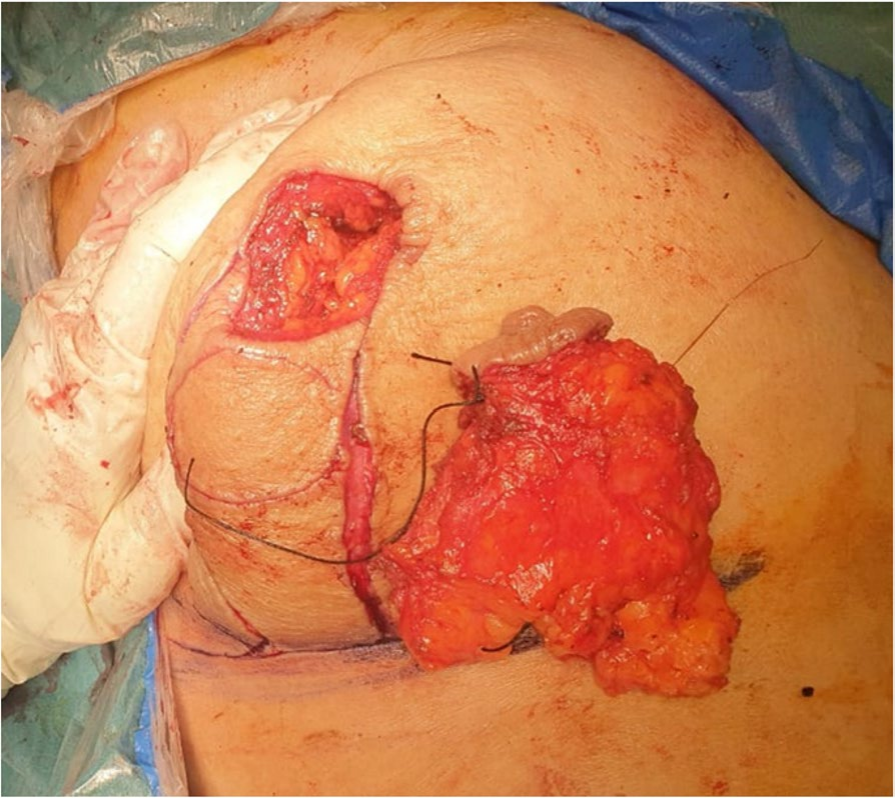
Central quadrantectomy including NAC and tumor with a cone of tissue

**Fig. 5:**
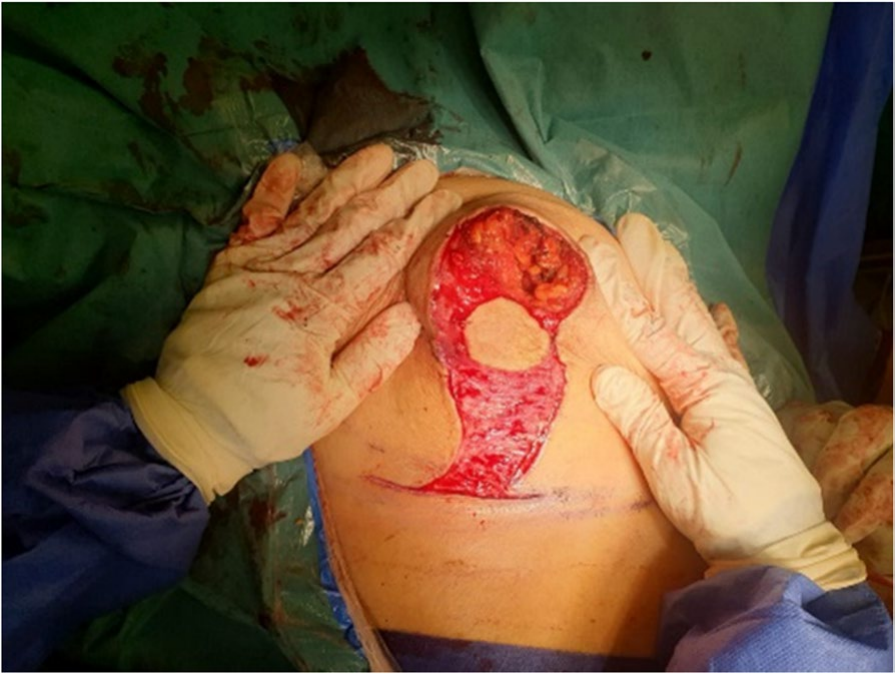
Complete de-epithelialization of the flap

**Fig. 6:**
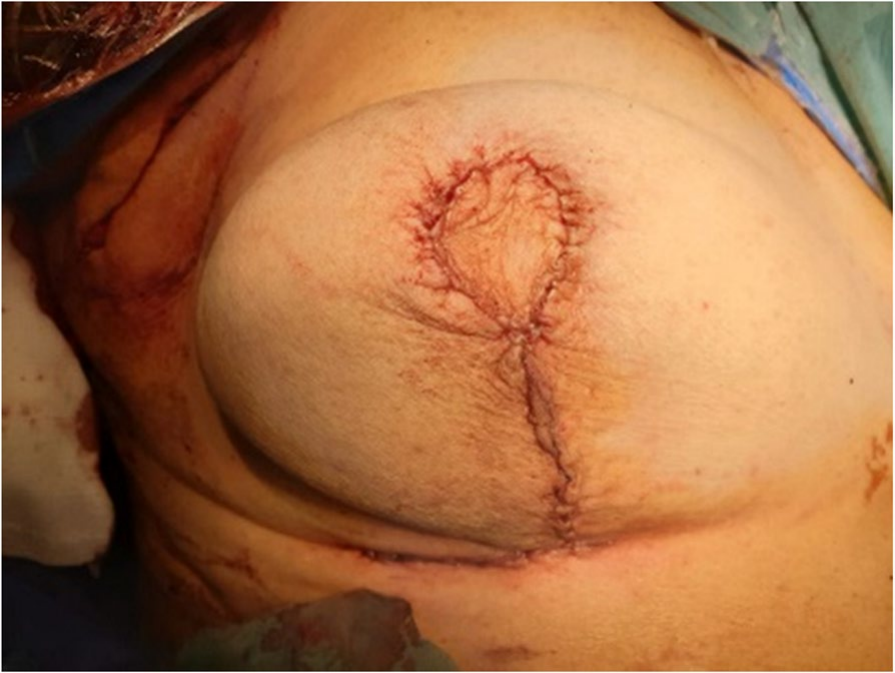
After closing the subcutaneous layers in Grisotti Flap mammoplasty

- The preoperative steps involve marking the inframammary line, NAC outline, new nipple and areola position below the NAC, and two vertical lines to mark the pillars of the flap, starting from the most lateral points of the old NAC and extending longitudinally down to the previously drawn inframammary line (Fig. 3).
- Next, a central quadrantectomy including the NAC and tumor with a cone of tissue from the subcutaneous layer down to the pectoral fascia was performed with ample safety margins (Fig. 4).
- Then, complete de-epithelialization of the flap was carried out, excluding the new areola (Fig. 5).
- A caudally located, de-epithelialized pedicled flap was created, including a skin island for restoring the areola (Fig. 6).
- After carefully and meticulously mobilizing the flap from the lateral pillars and dissecting down to the pectoral fascia, the flap was easily rotated upward to fill the empty central quadrant, with the skin island replacing the removed nipple-areola complex (Fig. 6).

**Fig. 7:**
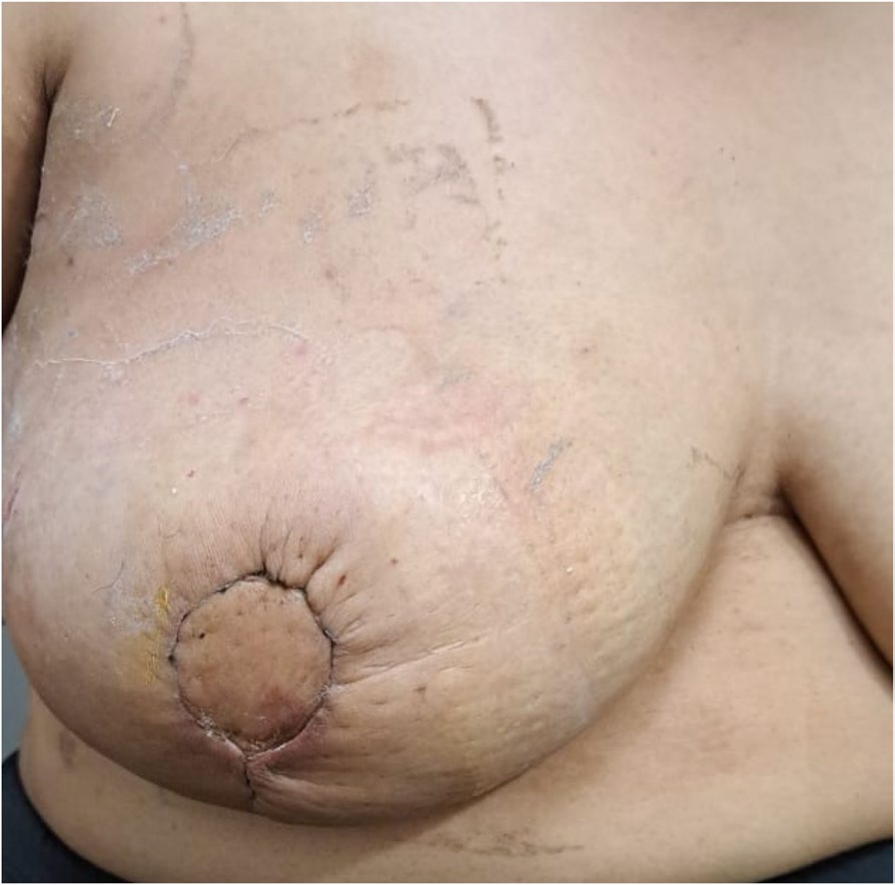
During early follow-up for the Grisotti Flap Mammoplasty group

During the year following surgery Fig. 7, patients underwent monitoring and management of potential complications, including wound infection, seroma, and recurrence. Oral antibiotics were administered for a week after the procedure, and wound dressing was performed on the second day postoperatively. All patients were instructed on the wound dressing process, and follow-up appointments were scheduled every two weeks until the wound was fully healed. Furthermore, follow-up appointments were held every two months for a period of two years after complete healing.

### Statistical analysis

The researchers utilized IBM SPSS Statistics version 26 to gather, process, and analyze the data. They transformed qualitative data into numerical values and percentages, while quantitative data was presented as means, standard deviations, and ranges with a normal distribution. For comparing two groups with qualitative data, the chi-square test was employed, and if any expected count in any cell was less than 5, the Fisher exact test was used. For comparing two independent groups with quantitative data and a normal distribution, the independent t-test was used, and for more than two groups, One-way ANOVA was applied. The confidence interval was set at 95%, and the margin of error accepted was 5%. Therefore, the *p*-value was considered significant as follows: *P* > 0.05: Not significant (NS), *P* < 0.05: Significant (S), and *P* < 0.01: Highly significant (HS).

## Results

Between May 2018 to May 2023, our Randomized control study was conducted on 40 patients, 20 patients in each group. Group(I) underwent nipplectomy and central quadrantectomy with areola preservation. While group (II) underwent the Grisotti procedure.

The mean age in years in the group (I) is 44.8 with a standard deviation ± 3.13. While in group (II) the mean age is 44.5 with standard deviation ± 4.85 (*p* = 0.848).

As regards co-morbidities of the patients 13(32.5%) were diabetic 6(30%) patients in group (I) and 7(35%) patients in group (II), 7(17.5%) patients were hypertensive3(15%) patients in group (I) and 4(20%) patients in group (II), 8(20%) patients were diabetic and hypertensive 5(25%) patients in group (I) and 3(15%) patients in group (II), 12(30%) patients had no co-morbidities 6(30%) patients in group(I) and 6(30%) patients in group (II) (*p* = 0.86) (Table 2).

**Table 2:** Shows the patients' characteristics

Characteristic	Group I (*n* = 20)	Group II (*n* = 20)	*P* value
Mean age in years ± SD	44.8 ± 3.13	44.5 ± 4.85	0.848
Co-morbidities (*n*)(%):			0.86
Diabetes	6(30%)	7(35%)	
Hypertension	3(15%)	4(20%)	
Diabetes and hypertension	5(25%)	3(15%)	
No co-morbidities	6(30%)	6(30%)	

Breast size, as reflected by bra cup size, was similar between the groups and ranged from cup sizes B to E. Tumor staging for both groups ranged from carcinoma in situ up to stage 3 carcinoma.

As regards the mean intra-operative time in minutes, in the group (I) was 80.1 with a standard deviation of ± 13.9, and ingroup (II) was 138.9 with a standard deviation of ± 14.02 (*p* = 0.001). Post-operative follow-up was done on all the patients to detect postoperative complications such as seroma, wound infection, patient satisfaction, recurrence after one year, and finally breast symmetry after two weeks and after a one-year follow-up. The seroma was detected in zero cases in group (I) and 2(10%) cases in group II (*p* = 0.487) and those two cases were managed by aspiration only. Regarding, wound infection, it was found in one case (5%) in group (I) and 3(15%) cases in group II (*p* = 0.605), and those cases were managed medically by oral and topical antibiotics along with anti-edematous drugs. The breast symmetry was detected after 2 weeks and after one year. Within two weeks the breast symmetry was detected in 20(100%) cases in group (I) and zero (0%) cases in group (II) in the form of a decrease in one cup size (*p* = 0.0001). After one year of follow-up, the breast size decreased slightly in group(I) due to the radiotherapy and there was more decrease in group (II) (Table 3).

**Table 3:** Shows a Comparison between the two studied groups regarding intra-operative time and postoperative complications as the seroma, the wound infection, the patient satisfaction, the recurrence, and finally the breast symmetry in one-year follow-up

Variables	Group (I) *n* = 20	Group (II) *n* = 20	*p*-value
Intra-operative time Mean ± SD	80.1 ± 13.9	138.9 ± 14.02	0.001
Seroma (*n*)(%)	zero	2(10)	0.487
Wound infection (*n*)(%)	1(5%)	3(15%)	0.605

Postoperative patient satisfaction was assessed by the Likert scale (5-point scale). In which, 1-Very dissatisfied,2-Dissatisfied, 3-Unsure, 4-Satisfied, and 5-Very satisfied. In group (I) there were zero patients very dissatisfied. While in group (II) there were 2(10%) patients very dissatisfied (*p* = 0.48). In group(I) 2(10%) patients were dissatisfied. However, in group (II) there was 4(20%) patients were dissatisfied (*p* = 0.661). In group (I) only 1(5%) patient was unsure. While in group (II) there were 3(15%) patients who were unsure (*p* = 0.54). In group (I) 5(25%) patients were satisfied. While in group (II) there were 9(45%) patients satisfied (*p* = 0.32). Finally, In group (I) 12(60%) patients were very satisfied. While in group (II) there were 2(10%) patients who were very satisfied (*p* = 0.002). There was a statistically significant difference between the two groups as regards patient satisfaction.

As regards the recurrence, there were no cases of recurrence detected in the two groups within two years of follow-up (Table 4).

**Table 4:** Shows the Likert scale in evaluation of the patient satisfaction post-operative

	Very Dissatisfied (*n*)(%)	Dissatisfied *(n*)(%)	Unsure *(n*)(%)	Satisfied*(n*)(%)	Very Satisfied *(n*)(%)
Group(I) (*n* = 20)	0(%)	2(10%)	1(5%)	5(25%)	12(60%)
Group(II) (*n* = 20)	2(10%)	4(20%)	3(15%)	9(45%)	2(10%)
*p*-value	0.48	0.661	0.54	0.32	0.002
Total (*n*)(%)	2(5%)	6(15%)	4(10%)	14(35%)	14(35%)

Aesthetic outcome was evaluated by grading post-operative photographs from 5 independent reviewers, all of whom were plastic surgeons. Categories for evaluation included breast shape, breast symmetry, and breast volume. Each was given a score on a scale of 10 to 1 (9–10 Excellent, 8–9 very good, 5–7 good, 3–4 satisfactory, 1–2 poor). The mean aesthetic outcome results were significantly higher in group I compared to group II, with a mean score of 8.6 ± 1.8 in group I versus 5.5 ± 2.7 in group II for breast shape (*P* = 0.005), 7.9 ± 2.1 in group I versus 5 ± 2 for breast symmetry (*P* = 0.004) and 8.1 ± 2.3 in group I versus 7.05 ± 1.9 for breast volume in group II (*P* = 0.108).

## Discussion

In the oncoplasty era, Patient satisfaction is a major factor in the surgeon's decision-making. However, oncological safety even at the expense of the final aesthetic result will always be the major factor in the surgeon's decision-making. In this study, we attempted to find an answer for a technique with oncological safety and high patient satisfaction.

The mean age of the group (I) is 46.65 with a standard deviation of 6.6. While in group (II) the mean age is 45.85 with a standard deviation of 6.07 (*p* = 0.576).

As regards the mean intra-operative time in minutes, in the group (I) was 80.1 with a standard deviation of ± 13.9, and ingroup (II) was 138.9 with a standard deviation of ± 14.02 (*p* = 0.001).

Regarding postoperative complications, they were significantly increased in the GFM group, 25% in the GFM group while 5% in CQ-NR-AP, which is greater than most other oncoplastic techniques for centrally located tumors mentioned in other studies which range 5–26% [7–11]. However, all cases of wound infection were treated medically and there was no need for any surgical intervention even cases of seroma were managed by ultrasound-guided aspiration, and only 1 in the GFM group had a delay in her adjuvant treatment for two weeks.

In the literature since GFM was introduced to us, the recurrence rate reported by authors has ranged, some reported zero recurrences as Moustafa and Fakhr [12] in 2014 with a follow-up period of 12 months while other studies [6, 10, 13] reported 2.3% to 9% recurrence rate with follow up period range from 6 to 76 month while in CQ-NR-AP group there are limited studies which discussed that technique with different results. Horiguchi et al [14] reported a 7.7% recurrence rate in 64 months of follow-up while Lino et al [15] didn’t mention any cases of recurrence. In our study, there were no cases of recurrence in either group.

Last but not least, patient satisfaction which was related to breast symmetry was considered to be one of the most significant differences between the two techniques in our study. As this point has not been extensively discussed previously, it is important to note that patient satisfaction has a great impact on the surgeon's preference. Patient satisfaction was assessed after one year of follow while breast symmetry was assessed at two points early after two weeks post-operative and later one year after and it was observed that breast size in all groups was reduced in size in the later assessment mostly due to radiation effect. In our study, patient satisfaction was observed to be higher in the CQ-NR-AP which relies on better breast symmetry and areola preserving which has a great impact on the aesthetic results. According to most studies [6, 12, 16, 17] patients with excellent satisfaction in GFM range between 70 to 90%, while CQ-NR-AP showed great patient satisfaction which was much better than the other group with better breast symmetry at all points of comparison, As Lino et al [15] stated that cosmetic result of Breast-conserving surgery with nipple resection was excellent in all patients.

## Conclusion

Central quadrantectomy and nipple resection with areola preservation have been found to be safer and more straightforward, with a lower incidence of complications compared to Grisotti flap mammoplasty, according to a two-year follow-up. Moreover, this study recommends using CQ-NR-AP as a popular and reliable procedure to be incorporated as a key operation in various classic procedures for cases involving central breast lesions that extend to the nipple. The goal of improving patient satisfaction is one of the most challenging aspects of managing centrally located breast tumors, and this study suggests that the CQ-NR-AP procedure may be a viable option.

## Data Availability

This is a prospective study including 40 patients presented with centrally located breast cancer divided into two groups each group consisting of 20 patients. Group I was subjected to nipplectomy and central quadrantectomy with areola preservation and Group II was subjected to Grisotti flap mammoplasty. The study was done from May 2018 to May 2023 including a one-year follow-up postoperative. The datasets used and/or analyzed during the current study are available from the corresponding author upon reasonable request.

## Abbreviations

CQ-NR-AP: Central quadrantectomy and nipple resection with areola Preservation
GFM: Grisotti flap mammoplasty
NS: Not significant
S: Significant
HS: Highly significant
ECG: Echocardiography
MRI: Magnetic resonance imaging
